# Addressing the Younger Age at Onset in Breast Cancer Patients in Asia: An Age-Period-Cohort Analysis of Fifty Years of Quality Data from the International Agency for Research on Cancer

**DOI:** 10.1155/2013/429862

**Published:** 2013-09-02

**Authors:** Seyed Houssein Mousavi-Jarrrahi, Amir Kasaeian, Kamyar Mansori, Mehdi Ranjbaran, Mahmoud Khodadost, Alireza Mosavi-Jarrahi

**Affiliations:** ^1^Department of Biostatistics, School of Public Heath, Mashhad University of Medical Sciences, Mashhad, Iran; ^2^Department of Epidemiology and Biostatistics, School of Public Health, Tehran University of Medical Sciences, Tehran, Iran; ^3^Department of Epidemiology, School of Public Health, Shahid Beheshti University of Medical Sciences, Tehran, Iran; ^4^Faculty of Health Sciences, Simon Fraser University, 8888 University Drive, Burnaby, BC, Canada V5A 1S6; ^5^The Cancer Research Center of the Cancer Institute, Tehran University of Medical Sciences, Tehran, Iran

## Abstract

*Introduction*. There is an established fact that Asian breast cancer patients are, on average, younger than their European counterparts. This study aimed to utilize the data from the Cancer Incidence in Five Continents I through XIII (published by the International Agency for Research on Cancer) to examine what contributes to the younger age at onset in the Asian population. *Material and Methods*. Data (number of breast cancer cases and corresponding population figures) for 29 registries in Europe and 9 registries in Asia for the period of 1953–2002 was accessioned and pooled to form two distinct populations, Asia and Europe. The age specific rates were defined and analyzed cross-sectionally (period wise) and longitudinally (cohort wise). The magnitude and the pattern of age specific rates were analyzed using the age-period-cohort analysis. The constrained generalized linear model with a priority assumption of cohort effect as contributing factor to changing rates was used to analyze the data. *Result*. During the last 50 years, the rate of breast cancer increased for both populations with an estimated annual percent change of 1.03% (with 95% CI of 1.029, 1.031) for Asia and 1.016% (95% CI of 1.015, 1.017) for Europe. There were stronger cohort effects in the magnitude of rates among the Asian population compared to the European population. The cohort effects, expressed as the rate ratio with cohort born in 1970 as reference, ranged from 0.06 (95% CI 0.05, 0.08) to 0.94 (95% CI 0.93, 0.96) for Asians and 0.35 (95% CI 0.33, 0.36) to 1.03 (95% CI 1.02, 1.04) for Europeans. The estimated longitudinal age specific rates (adjusted for cohort and period effects) showed similar patterns between the two populations. *Conclusion*. It was concluded that a strong cohort effect contributes to the younger age at onset among Asian breast cancer patients.

## 1. Introduction

Breast cancer is a leading cause of mortality and morbidity all over the world. In 2008, close to 1.4 million cases were diagnosed with breast cancer worldwide [1]. The incidence varies among different populations with high rates seen in developed countries compared to developing countries [2, 3]. In general, breast cancer rates are highest in white European and lowest in east Asian populations [1, 4, 5]. The estimated incidence rate for women living in the south-east Asia region of World Health Organization' is 26.1 per 100000 population and this figure is 89.7 for women living in Western Europe [1]. The established risk factors of breast cancer are, mainly, early age at menarche, late age at menopause, nulliparity, number of live birth, and age at first live birth. Contrary to the large variations seen in incidence between population of Europe and Asia, the prevalence of the established risk factors is not very much different between the two populations [5, 6]. A higher risk of breast cancer among American and European women has been blamed for so-called “western lifestyle” characterized [7, 8] by the combination of early menarche, decreased parity, delayed childbearing, and a sedentary lifestyle. Studies of migrants have confirmed the relative importance of environment and lifestyle in the etiology of breast cancer [9–11]. The so-called “western lifestyle” is now very common in Asian countries such as Japan, Korea, Taiwan, and Hong Kong and is spreading fast in the economic booming region of East Asia. In addition to the major differences in magnitude of rates between Asians and Europeans, there is a distinct difference in the shape of age specific rates between the two populations. In Asian population, the age specific incidence curve peaks at 45–50 and then plateaus and even slightly decreases so that the rates after 60 years are less than or close to the rates at age group 45–55. In European population, the age specific curve increases steadily with no change of pace around age 50 and the increase continues up to age 80 with a peak around 65 years. A lower risk and the distinct pattern of age specific incidence rates among the Asian population (even in countries with great extent of similar lifestyle with western population such as Japan, Taiwan, and Hong Kong) have been a challenging issue among epidemiologists and cancer scientists to the extent that some have labeled breast cancer in the population of Asia as a different disease [12]. A recent symposium in Montreal, Canada, specifically addressing the same topic, highlighted the younger age at onset as one of the chief characteristics of breast cancer natural history in Asian population [12]. What contributes to the peculiar phenomenon of younger age at onset has been the subject of inertest to epidemiologists, and it has been hypothesized that a cohort effect among Asian population causes this phenomenon. The aim of this study was to use the incidence rates reported in the Cancer Incidence in Five Continents for the two populations of Asia and Europe in order to address the nature of the age specific rate differences between the two populations using age-period-cohort analysis.

## 2. Material and Methods

### 2.1. Patients and Population

Registered cases of female breast cancer and corresponding person years were ascertained from the *CI5plus, Cancer Incidence in Five Continents Annual Dataset* (an online data repository of International Agency on Research on Cancer, IARC) for 29 registries in Europe and 9 registries in Asia for a duration from 1953 to 2002 [13]. Cases from Europe included cases registered for the period from 1953 to 2002 and cases from Asia included cases registered for the period from 1963 to 2002. Cases and their corresponding person years were pooled for each population to make two distinct populations, referred to hereafter as Asian and European.

### 2.2. Statistical Analysis

Descriptive and analytical approaches were used to analyse the data. Age specific rates were addressed both cross-sectionally (period wise) and longitudinally (generational birth cohort wise). For period wise age specifics, the rates were constructed and described based on five years period (1955, 1960, 1965,… 2000). For the cohort wise, age specific rates were estimated from age-period-cohort analysis constructed over each five years cohort. In addition, the trends in rates expressed as annual percentage change (APC) and their 95% confidence intervals were estimated using the age-period-cohort model.

For analytical part, the age-period-cohort model was used. For this, the periods and cohorts were constructed in intervals of 5 years. The period included 8 intervals for Asian population and 10 intervals for European population. The cohort included 19 intervals for Asian population and 21 intervals for European population. The five-year age groups were truncated to age more than 25 years with the last interval (85 and over) included all cases more than 85 years (13 five-year age groups were constructed). The constrained generalized linear model (CGLM), the most utilized approach in the epidemiology literature dealing with age-period-cohort analysis, was used. For this purpose, a log-linear model with the general form that includes *a* (age), *p* (period), and *c* (cohort) was applied as follows:

(1) Log{λ(a,p)}=f(a)+g(p)+h(c),

where *a*, *p*, and *c* represent the mean age, period, and cohort and *f*, *g*, and *h* are parametric functions fitted to the data. In this model, in addition to estimating the main effect of age, other components contributing to magnitude of rates specially the secular changes of rate across study periods and birth cohorts are estimated. The secular change or net drift corresponds, interchangeably, to hazard due to period or cohort, and it has been used to estimate the annual percent changes of rates over a period of time [14, 15]. As the purpose of our study was to tackle the difference between the two populations' age specific rates, it was assumed that mainly the cohort effect explains the changing of rates across aging intervals during the study period in both populations. With this assumption, the model estimates the age function presented as the log of the age specific rates for the reference cohort (longitudinal age specific or age specifics across cohorts) and the cohort effect as log of rate ratio relative to a reference cohort while period effect constrained to be zero on average with zero slopes. The estimated logs of age specific rates were transformed to rate scale (number per 100000 population) for better realization. In the model, the cohort born during 1970 was considered as the reference cohort and the period of 1970 was considered as the reference period. The longitudinal age specific raters were estimated and reported for cohort born on 1885, 1910, 1930, 1950, and 1970. For details of the modeling please refer to “age-period-cohort models for the Lexis diagram” by Carstensen [16]. Data were analyzed using the R 2.14.1 statistical software utilizing Epi 1.1.9 package (R Development Core Team, 2009).

## 3. Results

A total of 236,851 cases of breast cancer registered in the 29 European registries and a total of 188,630 cases registered in the 9 Asian registries were included in the analysis (Table 4 presents details of the included registries for the two populations).

### 3.1. Descriptive Approach

**Figure 1:**
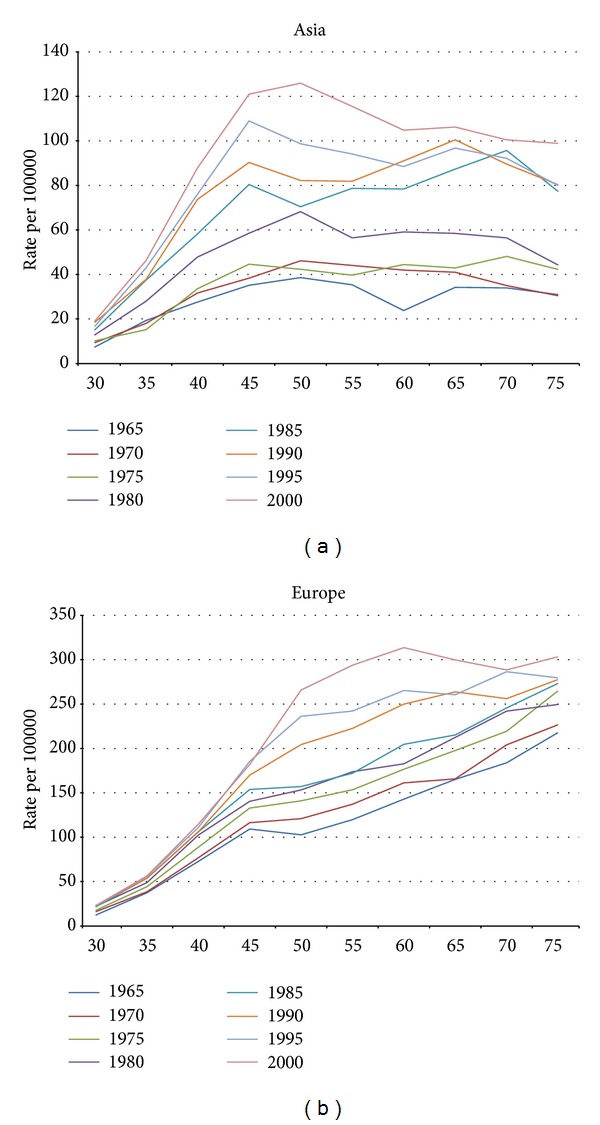
The cross-sectional age specific rates for both populations.

There was a constant increasing of rates for both populations during the last 50 years with an estimated annual percent of change 1.03 (with 95% CI of 1.029, 1.031) for Asians and 1.016 (95% CI of 1.015, 1.017) for Europeans. The incidence rates across all age groups in Europeans were higher than Asians, especially in older age groups. During the study period, the magnitude of rates increased for both populations for each succeeding five-year period for all age groups. The shape of the age specific rates (period wise) showed basic differences between the two populations. For Asian population, the age specific rates for all periods peaked around 50 years and then decreased and plateaued afterward (Figure 1). For the European population, the age specific rates increased up to the last age group for periods ending 1985 and for the periods after 1985, the age specific rates peaked between 55 to 75 years and then slightly decreased (Figure 1).

**Figure 2:**
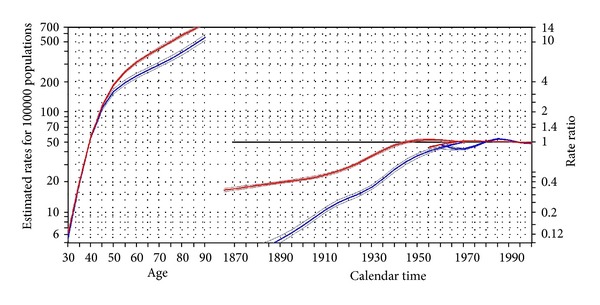
Estimated effects and their 95% confidence intervals from the age-period-cohort model (breast cancer, age group >25 years, period 1953–2002, and cohort born on 1865–1970 for both European (red) and Asian (blue) populations). Curve in the left represents the estimated age specific rates for the reference period 1970. The middle curve shows the rate ratios of cohort relative to the reference cohort (1970), cohort effect. The rightmost curves show the rate ratios of period constrained to be zero on average with zero slopes or the residual period effect.

**Figure 3:**
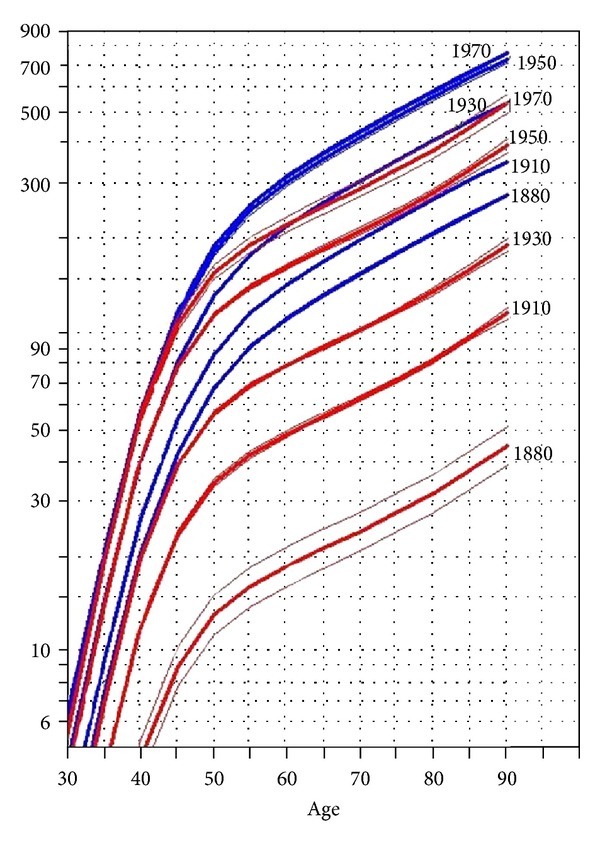
The graphical presentation of the estimated rates for different generations for both populations (red for Asian and blue for European).

The fitting of the age-period-cohort model to data indicated that the model that included all the main effects (age, period, and cohort) has the greatest reduction of deviance, indicating the best model to explain the observed rates in both populations; Table 1 presents the goodness of fit of the models along with their parameters. There were cohort effects present in incidence rates of both populations during the study period; however, the cohort effects in Asians were much stronger than European. In the Asian population, the rate ratios presenting the cohort effects ranged from a low of 0.06 (95% CI 0.05, 0.08) for those born in 1870 to 0.94 (95% CI, 0.93, 0.96) for those born in 1965. In the European population, the rate ratios presenting the cohort effect ranged from 0.33 (95% CI, 0.32, 0.35) for cohort born in 1865 to 1.03 (95% CI, 1.02, 1.04) for the cohort born in 1965 (Table 2 and Figure 2). There were residual period effects in the Asian population around 1975 (rate ratio of 0.89 and 95% CI 0.86, 0.92) and 1985 (rate ratio of 1.12 95% CI of 1.09, 1.13), Figure 2. For both populations, the estimated age specific rates expressed as longitudinal age specific indicated the same pattern for both populations; the age specific rates increased sharply before the age of 50, and the increase slowed down pace with the last age groups (over 75 years), still the groups with highest incidence rates. The pattern of longitudinal age specific rate is presented in Figure 2 along with the other effects, cohorts, and periods. The estimated longitudinal age specific rates (in an increment of 20 years) and their corresponding confidence intervals are presented numerically in Table 3 and graphically in Figure 3. As Table 3 and Figure 3 indicate, the estimated age specific rates have steadily increased in all age groups for both populations but the increase is more in Asians compared to Europeans. There is a large difference in the magnitude of rates between the two populations in early cohort (1890) when they are compared with the most recent cohort (1970), Table 3 and Figure 3. The difference in age specific rates between early and late cohorts is indicative of the cohort effects that cause the distinct pattern of age specific rates observed between the two populations. In addition, comparing the magnitude of the cohort effects between the two populations (Figure 2), they indicate that, though, the cohort effects are decreasing along succeeding cohorts for both populations, but the decrease in cohort effects in Asians is far larger than those of Europeans. This difference in decreasing rates of Cohort effects between the two populations indicates that both populations may experience similar rates in the future if there are no other major changes to the underlying cause of the disease in future years. 

## 4. Discussion

Our study proved a steady increase of breast cancer rate with similar pace during the last 50 years for both populations. We demonstrated that there is no difference between the patterns of age specific rates between the two populations when rates are measured as longitudinal age specific rates. It was demonstrated that a strong cohort effect contributes to the differences in pattern of age specific rates between the two populations. The difference in breast cancer rates with low rates for Asians versus high rates for Europeans has been documented since registries in Asia started reporting population rates [17, 18]. While several studies have demonstrated marked differences in magnitude and the pattern of age specific rates among different countries of Europe and Asia, no study systematically and collectively has addressed the age specific rate differences in the two populations as our study did. An overall increasing trend of morbidity from breast cancer has been reported for all populations of the world and the increase has been attributed to ageing and increasing median age of women [15, 17, 19–22]. Our study showed a very similar increase of incidence between the two populations. This similarity in slope of increase indicates that despite the fact that the two populations are basically different in terms of culture, ethnicity, lifestyle, and social attributes, the breast cancer epidemic enforces its own pace of epidemic projection.

Any increase in incidence of breast cancer rates is due to either changing of risk factors or implementation of mass screening (especially mammographic screening). Either of the two can affect both magnitude and pattern of age specific rates. The difference in the pattern of the age specific rates between the two populations is well recognized and several studies have addressed this discrepancy; a study comparing the shape of the age specific rates between Taiwanese and Caucasian American reported that the age specific rates of breast cancer differed between the two populations and the study concluded that the difference is due to a cohort effect presented in Taiwanese [23]. Another study comparing breast cancer rates among populations of Singapore and Sweden attributed the difference in rates to a large cohort effect and concluded that this effect will decrease in future generations causing similar incidence rates between the two population in coming decades [24]. In addition to comparative studies, it has been demonstrated that the pattern of age specific rates for the populations of Japan, Korea, China, Singapore, Thailand, and Philippine has changed in the recent years attributing this change to changing of life style toward more westernization and implementation of mammographic screening [25]. The pattern and magnitude of age specific rates of breast cancer have been affected by screening mammography specially in the European population, and this effect has been mainly presented as increase of incidence in the age group of 50 to 70 [26–28]. This is compatible with our finding as it was demonstrated in Figure 1 that age specific rates for European population increased in that age group 50 to 70 and the change happened after 1985 when the wide spread use of mammographic screening started [27, 29]. Mammographic screening started in Asia in late 1990 [30, 31] and its effect on the shape of age specific incidence cannot be assessed in our study. Compatible with previous studies comparing the age specific rates of breast cancer between population of Europe and Asia, our study proved that a large cohort effect in Asian population rates plays a major role in the differences in age specific rates between the two populations. The age specific rates can be defined both cross-sectionally (period wise) and longitudinally (cohort wise). If there are no cohort or period effects, the two definitions will show similar magnitude and pattern of age specific rates. The distinct pattern of age specific rates (cross-sectional rates) between the two populations is in fact due to the cohort effect that was demonstrated in our study. In the other world, what contributes to the observed pattern of period wise age specific rates in Asian population is the additive nature of the cross-sectional definition of age specific rates in the presence of decreasing cohort effects. Since the rates for two sequential age groups come from two different cohorts, when the older cohort has lower risk compared to younger cohort, the cross sectional patterns of age specifics will decrease. The strong cohort effect that exists in the Asian breast cancer is responsible for a distinct pattern of cross-sectional age specifics seen in the Asian population.

In applying the CGLM model, the choice of constraint (period or cohort) is based on an external knowledge of the underlying cause of change of rates in a population. Our choice of cohort instead of period as constraint was based on previous studies that attributed the changes of the rates to a cohort effect [15, 19, 32]. The period effect has been mainly attributed when change of health policy (e.g., introduction of more sensitive detection techniques or availability of certain diagnostic procedures) causes the changing of rates. In the light of the nature of breast cancer risk factors that are mainly of hormonal and sociobehavioral nature, their change would translate on cohort effect than on period effect.

This study enjoyed data of adequate quality as the data were utilized from the registries that met acceptable degree of validity and reliability to be published in the International Agency in Research on Cancer (IARC) official report. In addition, the quality of the data for both populations is comparable owing to the efforts and quality assurances and control protocols that IARC requires for different registries contributing data to the Cancer Incidence in Five Continents reports; one may ask. We would have been able to draw the same conclusion on analyzing just the data of Asian population without the need of European population. The main reason for using European for comparison was that the fact that the specificity attributed to Asian breast cancer age specific rates has been defined as it has contrasted to the well-sestablished breast cancer epidemiology in Europe and western countries.

The methodology we used is a very established and routine way of analyzing rates at population level when the data of calendar time exist. The age-period-cohort analysis has been a major tool in the hands of demographers and, in recent decades well utilized by epidemiologists. The methodology, while very common in use, suffers major problem especially when it is utilized to attribute the underlying cause of changes of a rate to period versus cohort (the nonidentifiability problem). In our study, this problem was not a concern as our assumption was that the nature of risk factors in breast cancer would translate into cohort effect other than period effect.

## 5. Conclusion

It was concluded that no differences in the pattern of age specific rates exist between the two populations when the age specifics are measured cohort wise, and the difference seen in the period wise age specific rate is due to a strong cohort effect present in the Asian population rates.

## Figures and Tables

**Table 1:** Summary statistics of age-period-cohort model for breast cancer rates in European and Asian populations*.

Model	Asia			Europe		
	Df**	Deviance	*P* value	Df**	Deviance	*P* value
Age	514	11764.7		644	45047	
Age-drift	513	1920.7	0.000	643	8227	0.000
Age-cohort	509	1628.1	0.000	639	4809	0.000
Age-period	509	1546.2	0.000	639	8118	0.000
Age-period-cohort	505	1308.7	0.000	635	4377	0.000

*The model fitted sequentially.

**Degree of freedom.

**Table 2:** The magnitude of cohort effects for the two populations.

Year of birth	Rate ratio (95% confidence intervals)	
	Asia	Europe
1865	∗∗	0.33 (0.32, 0.35)
1870	0.06 (0.05, 0.08)	0.35 (0.33, 0.36)
1875	0.07 (0.06, 0.09)	0.36 (0.35, 0.37)
1880	0.08 (0.07, 0.10)	0.37 (0.36, 0.38)
1885	0.09 (0.08, 0.11)	0.38 (0.37, 0.40)
1890	0.11 (0.10, 0.12)	0.40 (0.39, 0.41)
1895	0.13 (0.12, 0.14)	0.41 (0.40, 0.42)
1900	0.15 (0.14, 0.16)	0.43 (0.42, 0.44)
1905	0.18 (0.17, 0.19)	0.45 (0.43, 0.46)
1910	0.22 (0.20, 0.23)	0.47 (0.46, 0.49)
1915	0.25 (0.24, 0.27)	0.51 (0.50, 0.53)
1920	0.28 (0.27, 0.30)	0.57 (0.55, 0.58)
1925	0.31 (0.29, 0.33)	0.64 (0.62, 0.65)
1930	0.36 (0.34, 0.38)	0.73 (0.71, 0.75)
1935	0.43 (0.41, 0.46)	0.83 (0.81, 0.86)
1940	0.54 (0.51, 0.57)	0.93 (0.90, 0.96)
1945	0.64 (0.61, 0.68)	1.00 (0.97, 1.03)
1950	0.73 (0.70, 0.77)	1.04 (1.02, 1.07)
1955	0.82 (0.78, 0.85)	1.06 (1.04, 1.08)
1960	0.88 (0.86, 0.91)	1.05 (1.04, 1.07)
1965	0.94 (0.93, 0.96)	1.03 (1.02, 1.04)
1970	1.00	1.0

**No data for Asian population.

**Table 3:** The estimated longitudinal age specific rates and corresponding 95% confidence intervals for the two populations for the cohort born in 1880, 1910, 1930, 1950, and 1970.

	Age group	1880	1910	1930	1950	1970
Europe	25	2.3 (2.2, 2.3)	2.9 (2.8, 3.0)	4.4 (4.3, 4.5)	6.3 (6.2, 6.5)	6.1 (5.9, 6.3)
	30	7.4 (7.3, 7.6)	9.5 (9.3, 9.7)	14.5 (14.3, 14.8)	20.9 (20.6, 21.1)	20.0 (19.6, 20.5)
	35	20.6 (20.3, 20.9)	26.4 (26.1, 26.7)	40.4 (40.0, 40.8)	58.0 (57.6, 58.5)	55.6 (54.2, 57.0)
	40	42.6 (41.9, 43.2)	54.5 (54.0, 55.0)	83.5 (82.8, 84.2)	119.9 (119.2, 120.7)	114.9 (111.8, 118.1)
	45	68.2 (67.3, 69.2)	87.4 (86.7, 88.0)	133.8 (133.0, 134.6)	192.2 (191.0, 193.3)	184.1 (178.9, 189.5)
	50	92.3 (91.0, 93.7)	118.2 (117.4, 119.1)	181.1 (180.1, 182.1)	260.1 (258.1, 262.1)	249.2 (241.9, 256.7)
	55	114.1 (112.5, 115.7)	146.1 (145.2, 147.0)	223.8 (222.8, 224.7)	321.3 (318.7, 324.0)	307.9 (298.9, 317.2)
	60	134.9 (133.1, 136.8)	172.7 (171.6, 173.8)	264.6 (263.3, 265.8)	379.9 (376.5, 383.5)	364.1 (353.5, 374.9)
	65	157.8 (155.7, 159.9)	202.0 (200.9, 203.1)	309.4 (307.9, 311.0)	444.4 (440.2, 448.6)	425.8 (413.5, 438.4)
	70	183.6 (181.3, 186.0)	235.1 (233.9, 236.3)	360.1 (357.9, 362.2)	517.1 (512.0, 522.2)	495.5 (481.1, 510.2)
	75	212.4 (209.7, 215.1)	271.9 (270.5, 273.4)	416.5 (413.5, 419.5)	598.1 (592.0, 604.3)	573.1 (556.4, 590.3)
	80	244.1 (241.2, 247.1)	312.6 (311.0, 314.3)	478.8 (475.2, 482.5)	687.6 (680.5, 694.8)	658.9 (639.5, 678.8)
	85	279.7 (276.1, 283.4)	358.2 (355.1, 361.3)	548.6 (543.0, 554.3)	787.8 (778.2, 797.6)	754.9 (732.1, 778.4)

Asia	25	0.4 (0.4, 0.5)	1.2 (1.1, 1.2)	1.9 (1.8, 2.0)	4.0 (3.8, 4.2)	5.4 (5.2, 5.7)
	30	1.6 (1.4, 1.8)	4.2 (4.0, 4.4)	6.9 (6.7, 7.1)	14.2 (13.9, 14.6)	19.4 (18.6, 20.2)
	35	4.6 (4.0, 5.3)	12.0 (11.7, 12.4)	19.9 (19.4, 20.3)	40.9 (40.3, 41.4)	55.6 (53.1, 58.3)
	40	9.0 (7.8, 10.4)	23.7 (23.0, 24.4)	39.1 (38.3, 39.9)	80.4 (79.5, 81.3)	109.5 (103.9, 115.4)
	45	13.1 (11.4, 15.1)	34.4 (33.5, 35.4)	56.8 (55.8, 57.8)	116.8 (115.4, 118.3)	159.1 (150.6, 168.2)
	50	16.1 (13.9, 18.5)	42.3 (41.2, 43.4)	69.7 (68.6, 70.8)	143.3 (141.0, 145.7)	195.2 (184.4, 206.7)
	55	18.7 (16.2, 21.5)	49.1 (47.9, 50.2)	80.9 (79.9, 81.9)	166.5 (163.3, 169.7)	226.7 (214.2, 239.9)
	60	21.3 (18.5, 24.5)	55.9 (54.7, 57.2)	92.3 (90.9, 93.6)	189.7 (185.3, 194.3)	258.4 (244.2, 273.5)
	65	24.2 (21.0, 27.8)	63.6 (62.4, 64.8)	104.9 (103.2, 106.6)	215.6 (210.4, 221.0)	293.7 (277.4, 310.8)
	70	27.6 (24.0, 31.8)	72.7 (71.4, 74.0)	119.9 (117.3, 122.5)	246.5 (239.8, 253.4)	335.7 (316.6, 356.0)
	75	32.0 (27.8, 36.8)	84.2 (82.6, 85.7)	138.9 (135.1, 142.7)	285.6 (276.9, 294.6)	388.9 (366.0, 413.3)
	80	37.7 (32.8, 43.4)	99.2 (97.3, 101.3)	163.7 (158.7, 168.8)	336.7 (325.4, 348.3)	458.5 (430.7, 488.0)
	85	44.9 (39.0, 51.8)	118.2 (113.8, 122.8)	195.0 (186.3, 204.1)	401.0 (382.2, 420.7)	546.1 (508.8, 586.1)

**Table 4:** Detail information about the included registries.

	Registry code	Registry name	Starting year of reporting data	Duration of contributing data (in years)
Europe	20800	Denmark	1953	49
	24600	Finland	1953	49
	25001	France, Bas-Rhin	1975	27
	25002	France, Calvados	1978	24
	25003	France, Doubs	1978	24
	25004	France, Haut-Rhin	1988	14
	25005	France, Herault	1988	14
	25006	France, Isere	1979	23
	25007	France, Somme	1983	19
	25008	France, Tarn	1983	19
	27603	Germany, Saarland	1970	32
	38002	Italy, Florence	1985	17
	38007	Italy, Parma	1978	24
	38008	Italy, Ragusa province	1983	19
	38009	Italy, Romagna	1988	14
	38010	Italy, Torino	1985	17
	38012	Italy, Lombardy, Varese province	1978	24
	38020	Italy, Modena	1988	14
	52802	The Netherlands, Eindhoven	1973	29
	57800	Norway	1953	49
	75200	Sweden	1958	44
	75602	Switzerland, Geneva	1970	32
	75605	Switzerland, St. Gall-Appenzell	1983	19
	82603	UK, England, Merseyside, and Cheshire	1975	27
	82604	UK, England, north western	1979	23
	82605	UK, England, Oxford	1985	17
	82609	UK, England, Birmingham, and west Midlands region	1979	23
	82610	UK, England, Yorkshire	1983	19
	82620	UK, Scotland	1975	27

Asia	34400	China, Hong Kong	1983	19
	39203	Japan, Miyagi prefecture	1975	27
	39206	Japan, Osaka prefecture	1963	39
	39208	Japan, Yamagata prefecture	1983	19
	60801	Philippines, Manila	1983	19
	70200	Singapore: Chinese	1968	34
	70200	Singapore: Malay	1968	34
	76401	Thailand, Chiang Mai	1968	34
